# New Trends in Photodynamic Inactivation (PDI) Combating Biofilms in the Food Industry—A Review

**DOI:** 10.3390/foods10112587

**Published:** 2021-10-26

**Authors:** Dan Wang, Emmanuel Kyere, Faizan Ahmed Sadiq

**Affiliations:** 1 School of Food and Advanced Technology, Massey University, Palmerston North 4410, New Zealand; paaemma.kyere@gmail.cn; 2 School of Food Science and Technology, Jiangnan University, Wuxi 214122, China; faizan_nri@yahoo.co.uk

**Keywords:** PDI, antibiofilm mechanisms, reactive oxygen species (ROS), hurdle strategies

## Abstract

Biofilms cause problems in the food industry due to their persistence and incompetent hygiene processing technologies. Interest in photodynamic inactivation (PDI) for combating biofilms has increased in recent years. This technique can induce microbial cell death, reduce cell attachment, ruin biofilm biomolecules and eradicate structured biofilms without inducing microbial resistance. This review addresses microbial challenges posed by biofilms in food environments and highlights the advantages of PDI in preventing and eradicating microbial biofilm communities. Current findings of the antibiofilm efficiencies of this technique are summarized. Additionally, emphasis is given to its potential mechanisms and factors capable of influencing biofilm communities, as well as promising hurdle strategies.

## 1. Introduction

Contaminations with foodborne pathogens may happen at any point in the farm-to-fork chain, due to unhygienic handling and exposure of food to environmental contaminants. Prevention of these contaminations is difficult due to multiple processing and marketing steps before consumption [1]. The presence of pathogenic bacteria has been a more rigorous challenge for certain foods like ready-to-eat products, minimally processed vegetables and fruits [2,3]. The ineffectiveness of microbial decontamination originates from multiple aspects, including incompetence of hygiene processing technologies, and/or induced resistant microbial cells by processing techniques such as ultrasonication, ultraviolet light, pulsed electric fields [4,5].

Most bacteria live in the biofilm matrix, where microbial communities colonize surfaces within self-secreted extracellular polymeric substances (EPS) [6,7]. Biofilm cells are recognized as 10–1000 times more resistant than planktonic counterparts [8], providing spoilage bacteria and pathogens greater resistance to hygienic treatments and threatening the food industry. Conventional antimicrobial processing that targets planktonic cells could be powerless due to the binded-cell aggregates and protection from gel-like matrix. Moreover, the ineffective treatment can induce more resistance of unaffected biofilm cells, incite the recurrence of microbial contaminations and even pathogen outbreaks [9].

A new technology that promises to meet these demands is photodynamic inactivation (PDI). PDI involves the use of a photosensitizer (PS) activated by visible light, leading to the production of reactive oxygen species (ROS) and consequently inactivation of microbial cells [10]. Inactivation of microbes is caused by ROS which has potential to deconstruct proteins, lipids and nucleic acids inside microbial cells and lead to cell death [11]. From the available literature, PDI has gained attention and popularity due to these characteristics [12,13]:

(1) It exerts effectiveness on deactivating a wide range of microorganisms encompassing bacteria, fungi and viruses;
(2) It exhibits the antimicrobial effect against various states of bacteria; of planktonic, biofilm and spore;
(3) PDI can eradicate microorganisms rapidly and resistance development is unlikely to happen in microorganisms;
(4) PDI at proper parameters generates limited influence on food commodities.

Notably, PDI can ruin biofilm structural and functional biomolecules while comparing with conventional antimicrobial techniques. It can decrease bacterial cell attachment, change matrix structures and even eradicate biofilms [14], demonstrating potential for using PDI to address biofilm challenges [15,16]. Some studies have examined its potential in preventing and/or eradicating biofilms in the food preservation and hygiene technique, and the interest in overcoming this microbial persistence is increasing.

Understanding the mechanisms, influencing factors and hurdle strategies of PDI in food scenarios will be critical to facilitate its applications in the industry and ensure microbiological food safety. This study addresses biofilm challenges in food environments and illustrates the potential of PDI used as the control strategy. Current findings of the antibiofilm efficiencies of this technique are summarized. Additionally, emphasis is given to its potential mechanisms and factors capable of influencing biofilm communities, as well as promising hurdle strategies.

## 2. Overview of PDI Application in Food

### 2.1. Fundamentals of PDI

PDI occurs when exogenous or endogenous photosensitizer [10] molecules are exposed to visible light, triggering the excitation of photosensitizer (PS) molecules. Excited PS is with a temporary higher energy state, when turning back to the ground state, it can collide with oxygen-containing molecules via two oxidative modes. The type I mode indicates that PS accepts electrons from molecular oxygen or a substrate, inducing the formation of a PS anion and a substrate cation. These ions react with surrounding molecules such as superoxide ion (O_2_^2−^), free hydroxyl radical (·OH) and hydrogen peroxide (H_2_O_2_) to yield ROS. The superoxide ion is most likely formed with molecular oxygen and promotes one-electron production from PS, free hydroxyl radical is formed connecting with H_2_O_2_ that can lead to the decomposition of lipids and proteins. The type II mode refers to the collision of PS and an oxygen molecule, alongside the production of singlet oxygen (^1^O_2_). Singlet oxygen has been claimed to have the greatest damaging potential of all ROS produced [17], which is rapid in eliminating and attacking the organic compounds it encounters.

Visible light comprises violet, blue, green, yellow to orange, bright red and dark red wavelengths. Visible light of certain doses is safe for humans and does not cause eye problems, skin damage, skin cancer or immune system suppression. Some visible light sources exhibit antibiofilm activities. Angarano, et al. [18] reported antibiofilm activities of violet (400 nm), blue (420 nm), green (570 nm), yellow (584 nm) and red (698 nm) light on *Pseudomonas fluorescens* and *Staphylococcus epidermidis*. It was found that violet and blue light irradiation successfully inactivated the bacterial communities, however, others (green, yellow and red lights) neither enhanced nor reduced the biofilm cell density significantly. Absorption wavelength of certain PS locates in certain region. For example, bacterial and fungal cells contain endogenous PS porphyrin absorb light in the range of 400–500 nm [19], curcumin absorbs at a wavelength of 420–580 nm [20]. Nevertheless, not all wavelengths of the light spectrum are commercial and applicable. Currently, reliable light sources for PDI involve laser, lamp and light-emitting diode [21]. LED has attracted much attention due to its bandwidth, long lifespan (50,000–100,000 h), low energy consumption and large area-irradiation [22]. The PDI dosage which represents the accumulated energy density (E) applied can be calculated as E = Pt, where P is the irradiance (power density) in W/cm^2^, and t is the time in seconds.

Generally, a chemical with the capacity to absorb photons and be excited to the electronic state is referred as PS. Endogenous photosensitizers (PSs) such as cytochromes, flavins, NADH, and porphyrins, are naturally present in the microbial cells [23]. Supplemental exogenous PSs have been shown to be more efficient in antibiofilm capabilities than only endogenous ones. A cationic porphyrin derivative was effective in removing *S. aureus*, *P. aeruginosa*, and *Candida albicans* biofilms while endogenous porphyrin was found to be ineffective [24]. Natural bioactive compounds and synthetic food additives are the most promising exogenous PSs to be applied in food environments which have been summarized in Table 1. Curcumin is applied as a food seasoning, hematoporphyrin is used for strengthening food nutrition, and hypericin is a naturally occurring pigment and a flavouring agent. Vitamins can be applied as PSs, such as riboflavin (vitamin B_2_), vitamin K_3_ and vitamin D, all of which are natural and safe [25,26]. Some synthetic PSs have been examined and found to be safe for their applications in the food environment; these include rose bengal (some countries have banned it), 5-aminolevulinic acid (ALA), erythrosine, Na-Chlorophyllin, TiO_2_ (functions as photocatalyst and photosensitizer simultaneously).

### 2.2. Current PDI Applications in Food

Visible light sources have been widely employed in food production and horticulture, crop cultivation, fisheries and poultry rearing, due to their advantages of alternatives to sunlight, non-thermal, easy-controlling, and low price. Visible light sources especially LEDs have been intensively studied for their positive roles in postharvest preservation such as delaying senescence, enhancing the nutritional quality of leafy vegetables, delaying or accelerating the ripening of fruits, preventing spoilage and inactivating pathogens [22,31,32,33,34].

PDI has shown microbial inactivation capability, especially in synergism with PSs, which has important implications for food hygiene and food preservation (Table 2). Apricots (*Prunus armeniaca*), plums (*Prunus domestica*) and cauliflower (*Brassica oleracea*) treated with hypericin and 585 nm light irradiation (6.912 J/cm^2^) showed a reduction on their surface counts of *Bacillus cereus* by 1.1, 0.7, 1.3 log CFU/g, respectively [35]. Apart from inactivation of pathogens on food surfaces, bacteria existing in liquid food, such as orange juice and skim milk, can also be reduced by this technique [36,37]. Biofilms involve a dynamic life cycle (attachment, microcolony formation and matrix production and dispersion) of surface-adhered microorganisms. Following attachment, colonized microorganisms are covered by extracellular polymeric substances (EPS), showing resistance against food hygiene treatments, 10–1000 times more than planktonic cells [38]. Chen, et al. [39] found that PDI treatment (5 min, 1.14 J/cm^2^) with 1.0 μM curcumin decreased *Vibrio parahaemolyticus* (~10^8^ CFU/mL) to non-detectable levels, while 20.0 μM curcumin with light irradiation (60 min, 13.68 J/cm^2^) eradicated *V. parahaemolyticus* biofilm. PDI treatment of 9.36 J/cm^2^ (30 min) irradiation and 50 μM curcumin decreased *V. parahaemolyticus* on cooked oysters from 5.2 to 3.7 log_10_ CFU/g; 100 μM curcumin and 9.36 J/cm^2^ (30 min) irradiation achieved the inactivation to an undetectable level [40]. PDI also indicated possibilities of utilization in detoxification, Temba, et al. [41] reported that curcumin-mediated PDI at 420 nm reduced *Aspergillus flavus* fungal spores for up to 3 log_10_ CFU/mL in suspension and 2 log_10_ CFU/g in maize kernels.

PDI has gained advantages as it maintains a balance between microbial decontamination and sensory attributes (color, flavor or texture) of treated food. It does not adversely induce changes in physico-chemical properties and nutritional qualities [31,43,44,45]. However, there are reports about adverse effects caused by inappropriate irradiance. For example Ghate, Kumar, Kim, Bang, Zhou and Yuk [32] reported negative color changes appeared during the preservation period when using PDI illumination on pineapples slices. It is worth to note that natural color of a PS can affect the appearances of food products. For example, curcumin, chlorophyll and riboflavin, they are more suitable for applications in dark-colored food. However, limiting the concentration of PS may help to reduce or eliminate the side-effects; for example, Wu, Hou, Cao, Zuo, Xue, Leung, Xu and Tang [45] demonstrated that the curcumin concentration lower than 10 μM did not influence color and flavor when applied to oyster.

## 3. Current Applications of PDI against Food Related Biofilms

The biofilm communities are ubiquitous in the food industry, whether on food products, equipment surfaces (e.g., conveyor belts, stainless steel processing benches, pipes), or packaging materials (e.g., glass, polystyrene). Microorganisms can colonize on plant surfaces as a result of the lengthy processing cycles, complexity of plant facilities, the availability of nutrients and ineffective hygiene treatments [46]. Biofilms contribute to the persistence of microbial contamination by shielding pathogens and spoilage bacteria from environmental stresses, acting as hot spots for horizontal gene transfer (HGT) of virulence genes, transforming previously benign strains into pathogens [47], and providing niches for antimicrobial-resistant mutagenic activities [48]. Thus, biofilms serve as vectors for transmission of microbial contamination. Biofilms pose great potential hazards for food safety and human health, which prompted the development of PDI technique.

### 3.1. Food Products

PDI has been studied for its disinfectant properties against biofilms grown on food products. Notably, some researchers investigated the effect of PDI on the reduction of microorganisms, which is distinct from the detection of biofilm cells. The distinction is that if detecting biofilms, food surfaces should be rinsed with sterilized distilled water or PBS buffer solution to remove planktonic cells. Attached cells on food products are biofilm cells; however, the EPS matrix formation is dependent on the microorganism incubation conditions (temperature, time length, etc.). For example, no EPS or three-dimensional biofilm was observed when *Aeromonas hydrophila* (~10^8^ CFU/mL) was inoculated on lettuce at 4 and 10 °C for 24 h; however, three-dimensional biofilms with dense cell aggregations were observed when the incubation temperature exceeded 15 °C. PDI has been used to decontaminate biofilms on pepper [49], whole milk [50], fresh salmon [51], and mandarin fruits [52] (Table 3).

### 3.2. Equipment Surfaces

PDI has been described as a “rapid, efficient and environmentally friendly” method of decontaminating equipment surfaces. The adhesion of microorganisms and biofilm formation involve stainless steel and rubber conveyor belt surfaces in the food industry [53]. Microbial intercellular porphyrin absorbs visible light at a wavelength of 405 nm. This illumination (26 mW/cm^2^, 4 h) reduced the viable *Cronobacter sakazakii* biofilm cells on 304 stainless steel coupons (48-h-old) by 2.0, 2.5, and 2.0 log at 25 °C, 10 °C and 4 °C, respectively [54]. The effect of PDI on *Listeria monocytogenes* biofilms (24-h-old) was investigated by Li, Kim, Bang and Yuk [51]; on stainless steel and acrylic coupons, an illumination of 748.8 J/cm^2^ decreased *L. monocytogenes* cells by 1.5 and 1.6 log_10_ CFU/cm^2^ respectively. In addition, the researchers assessed the disinfectant sensitivity of biofilms treated with PDI versus those without PDI treatment, they discovered that biofilms treated with PDI were more susceptible to disinfectants. This indicates the utility of PDI in conjunction with disinfectants on equipment surfaces.

It has been reported that PSs have been used to coat equipment surfaces. For example, metal nanoparticle-PS-based surfaces produced increased singlet oxygen during the PDI process; specifically, threefold increased singlet oxygen was produced from rose bengal close to Silver Island Films (SiFs), while glass sample without containing silver served as control. Not only in silver (Ag), nanoparticles of gold (Au), Zinc (Zn) and Nickle (Ni) are with similar characteristics [55]. Photocatalyst metal oxides, which have the ability to increase microbial intercellular oxygen content, have also been used in PDI. Yoon, et al. [56] reported that TiO_2_-coated stainless-steel surfaces reduced *Escherichia coli* attachment by 60–80% under fluid flow conditions. Similarly, nitrogen-doped TiO_2_ surfaces have been found to inhibit *E. coli*, *S. aureus* and *V. parahaemolyticus* colonization [57].

### 3.3. Food Packages

The use of PDI in food packaging has been investigated. McKenzie, Maclean, Timoshkin, Endarko, MacGregor and Anderson [16] used 405 nm light at a dose of 504 J/cm^2^ to reduce *E. coli* biofilms on glass and acrylic surfaces, achieving reductions of 7 and 5 log_10_ CFU/mL, respectively. Murdoch et al. [58] reported a reduction of 2.18 log_10_ CFU/mL of *E. coli* biofilms (99.8% inactivation efficiency) on agar surfaces when exposed to an average irradiance of 128 J/cm^2^ (405 nm).

The use of sodium-chlorophyllin (Na-Chl, 1.5 × 10^−7^ M) mediated illumination at 400 nm (20 mW/cm^2^, 15 min) decreased *L. monocytogenes* biofilms on polyolefin surfaces by 4.5 log_10_ CFU/mL. The efficacy of decontamination varied depending on strains. Under the same illumination parameters, the thermoresistant *L. monocytogenes* 56 *Ly* strain required a higher Na-Chl PS concentration (1.5 × 10^−4^ M) [59]. Silva, et al. [60] used 530 nm green light (10 mW/cm^2^) in combination with 500 μM erythrosine or 250 μM rose bengal to eradicate 48-h-old *S. aureus* biofilm after 30 min illumination. Rose bengal was found to be more effective than erythrosine at controlling biofilms due to more iodide numbers in the xanthene ring following light irradiation and its ability to produce nearly 100% singlet oxygen [61]. Within 5 min (0.54 J/cm^2^), curcumin (0.2 μM)-mediated PDI inactivated >99.99% of planktonic *L. monocytogenes* cells, and completely inactivated with 1.0 μM curcumin. The *L. monocytogenes* biofilms (72-h-old) were decreased from 1.95 to 1.40 (OD_600_) after 3.24 J/cm^2^ irradiation with curcumin at concentrations ranging from 5 μM to 20 μM. When the irradiation dose was increased to 6.48 J/cm^2^ the OD value decreased further to 0.93 in the presence of 20 μM curcumin [26]. *V. parahaemolyticus* planktonic cells were reduced to undetectable levels in 5 min irradiation (460 nm, 1.14 J/cm^2^) with 1.0 μM curcumin; however, a 20.0 μM curcumin exposure for 60 min (460 nm, 13.68 J/cm^2^) was required to achieve undetectable levels in *V. parahaemolyticus* 48-h-old biofilms. They observed that curcumin-mediated PDI successfully downregulated virulence genes (*tdh* and *toxR*) and biofilm formation related genes (*oxyR*, *aphA*, *luxR* and *opaR*) [39].

## 4. Potential Mechanisms of PDI Antibiofilm Activities

PDI has a strong effect on inhibiting biofilm formation and dismantling established biofilms, and the underlying mechanisms are complex. Rapid ROS generation and photocatalytic oxidation are presumed to play predominant roles in PDI. They can disrupt the balance of the microbial community physiology, lead to biofilm cell lysis and biofilm matrix structure disruption [62,63]. Understanding the physiological responses of biofilm communities and their genotypic expressions are critical for PDI development (Table 4).

### 4.1. ROS and Cell Lysis

ROS, which include oxygen radicals, singlet oxygen and peroxides, exist naturally in microorganisms as signaling molecules that retain cells in a redox physiological state [68]. Once the disturbance manifests, the accumulation of excess ROS is referred to as oxidative stress. Excess ROS can be generated as a result of multiple antimicrobial treatments, changes in the pH or other environmental stresses [69,70]. Following, the cells initiate a redox defense mechanism against the oxidative stress, which includes promoting EPS matrix production and adjusting biofilm heterogeneity to enhance stress tolerance. However, if ROS are generated and accumulated in a rapid manner and the intracellular redox defense mechanisms fail to cope with the stress, the excess ROS will cause lipid peroxidation, cell membrane damage, nucleotide degradation and eventually cell death [71,72].

DNA oxidation alters the nucleoid and ribosome areas within the microorganism, impairing genomic functions. This effect caused by PDI illumination in *Salmonella enteritidis* and *Salmonella* Saintpaul was observed through transmission electrical microscopy [41] by Kim and Yuk [65]. Free radicals can react with DNA structural units, causing damage to purine and pyrimidine bases, as well as inducing additional DNA damage and double-strand fragmentation. The lipid peroxidation induced by the excess ROS occurs in polyunsaturated fatty acids (the predominant constituents of the fatty acid membrane), owing to their in-between methylene groups (-CH_2_-) and double bonds [64]. The methylene groups render hydrogen atoms in the cell membrane, making it more susceptible to ROS destruction. The degradation of lipids is a self-propagating chain reaction, and the initial oxidation can result in programmed cell death—apoptosis. Apoptosis is characterized by cell shrinkage, plasma membrane blebbing and nuclease activation. Apoptosis has been identified as the main form of cell death in response to PDI [73]. PSs binds to lysosomes, mitochondria, and/or other intracellular membranes, all of which are where apoptosis pathway reactions occur [74]. In contrast to apoptosis, necrosis (non-programmed death) is characterized by random DNA fragmentation, cell swelling, lysis, and the induction of an inflammatory response. Cells may die due to apoptosis or necrosis caused by PDI. This may depend on the presence of the needed enzymes responsible for apoptosis such as caspases and the Bcl-2 family members [75].

### 4.2. Elimination of Biofilm Matrix Molecules

The two most prevalent damaging ROS (·OH, free hydroxyl radical and ^1^O_2_, singlet oxygen) can react with a wide range of biomolecules. The most highly reactive compound produced by PDI is singlet oxygen [76,77]. On one side, the singlet oxygen attack is non-specific to all non-cellular biomolecules, where the singlet oxygen can interact with biofilm matrix compounds and cause compound reaction [78]. Besides that, antioxidant enzymes (e.g., peroxidase, superoxide dismutase, catalase) protect against some other ROS, but not against singlet oxygen [77]. Proteins are degraded through oxidation of Try/Met/His residues, meanwhile, lipids are per-oxidized, water is hydrogen abstracted, and biofilms polysaccharides are equally susceptible to photodamage, resulting in EPS matrices breakdown. According to Davies [79], the efficiency of the singlet oxygen reaction varies with compound composition of biofilms: protein 68.5%, lipids 0.2%, DNA 5.5%, RNA 6.9%, NADH/NADPH 0.6% (NADPH, reduced form of nicotinamide adenine dinucleotide phosphate).

### 4.3. Intervention of Cell Motility and Quorum Sensing

Though PDI is designed at lethal doses, pathogens may encounter sublethal doses during treatment. Sublethal PDI dose cannot inactivate microorganisms, but it has been shown to influence motility and quorum sensing (QS), both of which are required for mature biofilm formation [80].

Motility is the very first step of the bacterial biofilm formation life cycle, and flagellar functions to sense the environmental signals and produce motility forces. The flagellar of *Vibrio vulnificus* was reported to be degraded immediately after PDI treatment (toluidine blue as the PS) while the rest of the cell remained intact. Increasing treatment time to 2 min, and the PDI severely damaged the cells; however, the pili (responsible for swarming motility) remained intact [81]. It has been reported that treating the cells with a low PDI dose did not fragment the flagellar but rather caused the flagellar to fly apart, with cells tumbling over one or more times [82]. The above findings could be attributed to the expression levels of relative genes; for example, it was discovered that the expression levels of flagellar motor gene (*oxyR*) were down-regulated by 13% in PDI illuminated *V. parahaemolyticus* biofilm cells [39]; similarly, gene *flgJ*, *fliD*, *flhD*, *motA* and *motB* showed a significant difference in expression after PDI treatment on *C. sakazakii* biofilm cells [54].

QS plays critical role in biofilm formation through cell-to-cell communication based on cell density, and it works through chemical secretory signals known as autoinducers (AI) [83]. Autoinducing peptides and autoinducer-2 (AIP and AI-2, in Gram-positive bacteria) and acyl-homoserine lactones (AHL, in Gram-negative bacteria) are the three categorized QS systems based on the structure and function of AI. Cell density, biofilm differentiation, and maturation are all regulated by QS, which suggests that QS regulation may play a role in biofilm challenges [84]. PDI can lower the microbial density, resulting in the lack of macromolecules required to initiate biofilm formation [85]. After being exposed to PDI illumination, the expression levels of QS genes decreased. The expression levels of QS related genes *abaI*, *agrA*, and *lasI* in *Acinetobacter baumannii*, *P. aeruginosa*, and *S. aureus* were downregulated to approximately 1.9-, 3.7-, and 4.9-fold, respectively [86]. PDI treatment down-regulated the expression levels of QS related genes in *Vibrio*, including *aphA*, *luxR* and *opaR*, by 9%, 30% and 12%, respectively [39]. The downregulation of the expression of QS-controlled biofilm formation genes (*pslA* and *pelF*) and QS genes (*lasI*, *lasR*, *rhlI*, and *rhlR*) were reported in *P. aeruginosa* ATCC 27853 [66].

### 4.4. Induction of SOS Response—A Cellular Response to DNA Damage

Sublethal PDI has been shown to increase DNA oxidation levels [87], oxidative stress response and SOS response [39,65,67]: *recA*, which is responsible for activating the SOS response and repairing DNA damage, was found of higher expressed in *S. enteritidis* and *V. parahaemolyticus* after PDI treatment. *OxyR*, *AhpC*, *GrxA*, *AtpC* and *SulA*, which are responsible for the oxidative stress response and SOS, were also found to be higher produced in PDI treated *S. enterica*. The oxidative stress and ROS response contribute to induction of SOS response, which inhibits cell division, initiates DNA damage repair or induces apoptosis like death [88].

## 5. Factors Influencing Antibiofilm Efficiency in the Food Industry of PDI

### 5.1. Light Engineering Variables

The antibiofilm effectiveness of PDI is dependent on laser engineering parameters such as light delivery rate and PS-to-light interval, which have been thoroughly validated and demonstrated in recent literature [89]. Regarding light delivery rate, high incident laser is not always better at antimicrobial efficiency. For example, when employing porphyrins as the photosensitizer, the light spectrum from 400 to 420 nm is preferred over others due to absorption performance of porphyrins [90]. Additional concerns include the PS-to-light interval, which some scholars refer to as pulsing. The reason for its contributions to efficiency is related to ROS production; however, the mechanisms remain unclear [91]. These two variables (light delivery rate and PS-to-light interval) are important since they determine PS-light product dose [92]. This suggests that the PDI kinetics and economic indexes should be combined, and considered in industrial applications [32].

### 5.2. The Structure and Dose of Photosensitizers

In light of cellular or biopolymer localization and antimicrobial efficacy, it is necessary to consider PS structures involving charge and polarity [93]. Polycationic PSs remain difficulty in penetrating the anionic biofilm matrix due to their strong binding. Besides, hydrophilic PS can bind with proteins and is hard to penetrate cell membranes. This also means that using hydrophilic PS to disinfect meat-related environments may be difficult, as the cleaning efficiency may be compromised by the protein- and lipid-rich conditions [23,94,95]. Encapsulation or PS modification can be used to adjust PS characteristics, enabling the most versatile performance possible for PDI. When PS was functionalized by attaching it to bioactive nanoparticles, its affinity for the cell membrane was increased, allowing for greater penetration into the biofilm structure [96]. Biofilm removal effect is dependent on the dose of photosensitizers, for example, the bio-film eliminations of *L. monocytogenes* were decreased from 1.95 to 1.40 (OD_600_) after curcu-min concentration was enhanced from 5 μM to 20 μM [26]. Similarly, 5 min irradiation (460 nm, 1.14 J/cm^2^) with 1.0 μM curcumin achieved to inactivate *V. parahaemolyticus* planktonic cells, once increased the PS dose to 20.0 μM, the PDI could eliminate 48-h-old *V. parahaemolyticus* biofilms cells [39]. However, the effects of PS dose on food sensory and toxicity should be further studied.

### 5.3. Mono/Multispecies Biofilms

Teixeira et al. [97] reported PDI treatment (55 J/cm^2^) successfully reduced *Streptococcus mutans* mono-biofilm in vitro but did not inhibit multi-species biofilm growth. A similar observation was also obtained from Shany-Kdoshim et al. [98]. The coaggregation and metabolic cooperation of neighbouring species within the biofilm matrix is the reason for this scenario [99,100]. Some researchers, however, argued that other factors, co-residence and strain variations, might account for this observation. Some others claimed that some bacteria were in a competitive or antagonistic relationship, which could decrease the biofilm matrix stability [101]. For example, *P. aeruginosa* appeared to increase the sensitivity of biofilm to chloroxylenol when grown with *S. aureus* [102].

### 5.4. Biofilm Thickness and Structures

PS of over 50 nm in diameter, will be difficult to diffuse into the thick and well-aggregated biofilm cell to commence the photodynamic inactivation [103]. Teixeira et al. [97] studied the photodynamic effect (638.8 nm, 40 mW/cm^2^, 15 min) in combination with toluidine blue (100 mg/L as PS) on in situ and in vitro *S. mutans* biofilms. The CFU of in vitro biofilms was reduced by more than 5 log_10_/mL, whereas that of in situ biofilms was reduced by less than 1 log_10_/mL. This was due to the thickness of in situ biofilms, which was 10-fold thicker than in vitro biofilms, and toluidine blue was difficult to penetrate inside the biofilm matrix.

The biofilm architecture consists of towers, mushroom-like structures and monolayer shapes with varying stabilities against various environmental stresses [104,105]. Monolayered biofilms have been found to form in cold temperatures (4 and 10 °C) which are poorly structured [106]. Inoculation density and nutrient level determine whether biofilms will be mushroom-shaped. The mushroom-shaped biofilms are difficult to combat because they have an immotile attachment base and a motile cap [106]. Notably, biofilm matrices that survive disinfection can re-establish and readjust their metabolic activities to restore bacterial viability.

### 5.5. Contact Surfaces

The texture, hydrophobicity, and charge of the substratum surface influence cell adhesion, colonization and biofilm formation. Surface topography, such as scratches and cracks, promotes biofilm formation and increase difficulties in removing biofilm [107]. Stainless steel is widely used as the food processing equipment surface due to its durability, ease-of-cleaning and resistance against corrosion. Grade 316 Stainless steel is the most resistant to chemical treatments such as sodium hypochlorite and is therefore less prone to deterioration than other stainless steel grades. The food industry also uses stainless steel of grade 304, but it is not resistant to chemical cleaners and sanitizers. For transportation and processing in the food industry, a variety of containers and cutting boards are used, including rubber, expanded polystyrene, fibreboard, polypropylene boxes, and wood [53]. PDI efficiency on different contact surfaces should be investigated further.

### 5.6. Food Environments

Food residues, protein-rich, lipid-rich and carbohydrate-rich, have been identified to protect pathogens from stressful conditions [108,109]. Food residues may negatively influence antibiofilm efficacies of PDI. According to McKenzie et al. [16], higher log reductions of *L. monocytogenes* biofilms on glass surfaces were achieved without salmon meat as the inoculum nutrition, indicating that food substratum would favor *L. monocytogenes* recovery from stressed conditions, thereby impairing PDI disinfection effect. Kuda et al. [110] reported that food residues, such as 10 μL of meat (beef, pork) gravy and milk, and 1 μL of egg yolk, hampered biofilm removal when *Lactobacillus sakei* and *Leuconostoc mesenteroides* were grown on a stainless-steel surface. pH values vary according to food environments. Curcumin-mediated PDI was more effective at killing *S. aureus* at pH 5.0 than at pH 7.2 or 9.0 [111].

## 6. Potential Hurdle Strategies against Biofilms

### 6.1. Organic Acids

Organic acids can improve the ecoefficiency of photodynamic disinfection. Ghate et al. [112] determined that three acids (lactic, citric, malic acids) with 461 nm LED illumination (22.1 mW/cm^2^) at 15 °C, inactivated *E. coli*, *Salmonella typhimurium*, *L. monocytogenes* and *S. aureus* within 4.5–7.5 h (PDI energy ranging from 358.0 to 596.7 J/cm^2^). After 7.5 h, they observed that citric acid reduced these four pathogens to non-detectable levels, and malic acid reduced all bacterial populations by approximately 3.5 log_10_ CFU/mL. The blank control demonstrated these acids did not show significant antibacterial effects (*p* < 0.05). Organic acids enhanced PDI inactivation efficiencies could be attributed to two mechanisms: their undissociated form, which help them easily penetrate the cell membrane and decrease intracellular pH; organic acids can also chelate metal ions on the cell membrane and disrupt cell structures, exposing cells to increased ROS exposure [113]. 7.5 mM and 10 mM ALA was used in conjunction with 400 nm light to investigate the synergistic effect on *L. monocytogenes* biofilm at relatively low light density and dose of 20 mW/cm^2^ and 24 J/cm^2^. The *L. monocytogenes* cells attached to polyolefin were reduced by 1.7 and 3.1 log_10_ CFU/mL, respectively [114]. ALA was recommended for use in food, even when in direct contact with food, due to its colourless, odourless and non-reactive nature with food commodities [115] (Table 5).

### 6.2. Nanobubble

Nanobubbles have gained much interest in broad fields including agriculture, food and medical [120]. Gaseous bubbles currently play critical roles in various food products to stabilize, to form microstructure and essential functions, including beverage, baked products (crackers, puff pastry, breads), dairy products (ice cream, cheese), chocolate and some confectionery products [121,122,123,124]. The gas used determines nanobubble characteristics. If more nanobubbles are expected, a greater solubility of the gas in the liquid is required. The stability of nanobubbles is dependent on the gas, with increasing order of oxygen > air > carbon dioxide [123]. Nanobubble formation techniques are being developed specifically for antibiofilm applications. The purpose of laser-induced vapor nanobubbles is to increase the amount of space between sessile cells. This technique utilizes expanding and collapsing water vapor generated by short (<10 ns) laser pulse, as well as a suspension of gold nanoparticles [125]. The air bubbles in water has been reported to be supportive of removing biofilms attached on stainless steel and polypropylene surfaces, as well as the carbohydrate, protein and fat residues in the food industry [126].

### 6.3. Ultrasound

Ultrasound is another technique that has been studied in conjunction with PDI. Ultrasound of low-frequency-high-power is considered a high inactive approach on microbes and biofilm, it can retain the sensory and nutritional quality of minimally processed fruit, juice and other food products [127]. Bhavya and Hebbar [128] studied ultrasound following 462 nm blue light (6.34 mW/cm^2^, 70 J/cm^2^) with and without PS (curcumin, 100 µM) on liquid food orange juice. Blue light and curcumin had the highest inactivation activities, resulting in *E. coli* and *S. aureus* reductions of 1.06 and 2.34 log_10_ CFU/mL, respectively. However, when ultrasound was used, it resulted in 4.26 and 2.35 log_10_ CFU/mL reduction, respectively. At optimized parameters, this hurdle had no adverse effect on the phenolic, total flavonoid and hesperidin content of orange juice markedly. This study suggests the possibility of combining PDI and ultrasound as a hygiene technique.

### 6.4. Biosurfactant

Biosurfactants are natural, low molecular-weight and surface interacted molecules encompassing hydrophobic and hydrophilic characteristics. It can inhibit bacterial attachment, weaken bacteria-bacteria interactions, and intervene biofilm formation by altering cell surface properties. Biosurfactants with antibiofilm activities have been investigated, especially those of low toxicity and possibilities to be used in the food industry. For example, Dusane et al. [129] found that glycolipid extracted from *Serratia marcescens* was able to inhibit adhesion of *C. albicans*, *P. aeruginosa* and *Bacillus pumilus* as well as disrupting preformed biofilms. V9T14 and V19T21 isolated from *Bacillus subtilis* and *Bacillus licheniformis*, were identified with outstanding antibiofilm activities. V9T14 reduced 97% and 90% *E. coli* CFT073 biofilm, respectively, by coating the polystyrene surfaces (2560 μg/mL) and adding it to the inoculum (10 μg/well); V19T21 hampered nearly 90% *S. aureus* ATCC 29213 biofilm formation when by coating of 160 μg/mL or by adding 5 μg/well [130]. BS-SLSZ2 was isolated from the marine bacterium *Staphylococcus lentus*, when using it at a dose of 20 g, *Vibrio harveyi* and *P. aeruginosa* biofilms were inhibited by 80 and 82%, respectively [131].

### 6.5. EDTA

The potential of using he cation chelator ethylenediaminetetra-acetic acid (EDTA) in PDI has been studied. Hu et al. [132] reported that EDTA significantly enhanced the antibacterial effect of PDI (curcumin as the PS) against *Burkholderia cepacia*. Without the addition of EDTA, 125 μM curcumin illuminated by 425 nm light (16 mW/cm^2^, 30 min) induced 40% cell death. Combined with 0.2% (*w*/*v*) EDTA and 50 μM curcumin, the viability of *B. cepacia* was only 7.57%; while with 0.4% (*w*/*v*) EDTA, the population was almost eliminated (>4 log_10_ CFU/mL reduction). The SEM observation demonstrated that the combinational treatment resulted in irreversible cell wall damage, leakage of the cytoplasmic compounds and finally, degradation of DNA and protein. The potential mechanism was the disruption of the lipopolysaccharide in the outer membrane [117]. Polysaccharides are neutral or polyanionic in Gram-positive biofilms, while in Gram-negative biofilms, cationic type is prevalent [48]. EDTA can chelate the divalent cation (calcium, magnesium, iron) which are essential to the stability and maintenance of biofilm matrix, and thus achieve biofilm removal [133]. EDTA has shown antibiofilm activities against *S. aureus* and *P. aeruginosa* biofilm, which induced biofilm dispersal [134]; however, its synergistic antibiofilm effect with PDI is still lacking.

### 6.6. Nanocarrier Particles (NPs)

Antibiofilm effect was promoted by combining PSs with nanocarrier particles [131]. This has been attributed to reduced efflux of PS, inhibition of PS degradation, controlled release rate and resultant production of ROS [96,135]. For example, photoactive metallated porphyrin-doped conjugated polymer nanoparticles (CPN) were conducted to evaluate the antibiofilm capabilities [14]. It was found that up to 3.01 log_10_ CFU/mL reduction was observed using 16.5 mg/L CPN within the PDI illumination. CLSM images confirmed the disruption of the biofilm matrix and cell death from CPN-PDI treatment. Outstanding NPs for antibiofilm strategies allow interaction with both biofilm extracellular polymeric substances and bacterial cells. For example, chitosan (poly-[1,4-b-D-glucopyranosamine]) initiates with the NP attachment on the biofilm then proceeds to enable electrostatic interaction between positively charged nanoparticles, and the negatively charged bacterial surface [96,136]. The interaction between NPs and biofilms can be enhanced by the size of NPs, which can lead to more NP diffusion into the biofilm matrix and greater possibilities to kill bacteria. Additionally, the positive charge and smaller size of NPs can increase its diffusion coefficients [137].

### 6.7. Essential Oils

Essential oils have been known for their significant antibacterial activities, while some of them have demonstrated antibiofilm activities. For example, essential oils from *Cinnamomum tamala* has been reported that inhibited the expression of flagellum, cytotoxins and biofilms [138]. Citral is a key component in essential oils from citrus peel, and it has been shown to inhibit *V. parahaemolyticus* biofilm formation by repressing transcription of flagella, type III secretion, toxin and quorum sensing related genes [139]. According to Marques-Calvo et al. [21], eucalyptus (*Eucalyptus globulus*), clove (*Eugenia caryophyllata*), and thyme (*Thymus vulgaris*) essential oils enhanced the antibacterial effect when combining with visible light. However, the antibiofilm effects of the synergistic treatments combining PDI and essential oils remain knowledge gaps.

### 6.8. Chemical Disinfectants

Chemical disinfectant washing can be applied as a hurdle technique defending against biofilms with PDI. Chlorinated disinfectants are widely applied in the food industry; among them, sodium hypochlorite (NaOCl) is easy to access and always compatible with other detergents. Kwan [140] found that 5 min blue light (440 nm) irradiation while in combination with NaOCl obtained increased reduction of *Enterococcus faecalis* biofilm adherence. Other potential disinfectant applications involve electrolyzed water and cold plasma treated water, which exhibit antibiofilm capacities and are considered as hurdles. Acidic electrolyzed water (pH 2.28) can eliminate key structural EPS components of the *V. parahaemolyticus* biofilm and prevent its reestablishment [141]. Cold plasma-activated water inactivates microorganisms while retaining nutritional food content [142]; the disinfection capacities are due to ROS and reactive oxygen and nitrogen species (RONS) from ionized neutral gas [143,144].

## 7. Future Perspectives and Conclusions

PDI has potential for preventing and eradicating biofilms in food-related environments, as it is a sustainable technique that does not induce microbial resistance, and is environmental-friendly. However, scaling up of PDI for industrial applications demands some aspects to be addressed. Other conditions, such as batch systems for processing large amounts of food and materials, must be investigated. One of the other concerns is the limited penetration depth of visible light. Micro- and millimeter-scale penetration of light through food or other substrata results in problems with limited applicable conditions of PDI [19]. Further exploration is required to expand the suitability of PDI on various applicable scopes.

The performance of PDI is attributed to many factors, including light laser variables, PS structures and dose, biofilms formed by mono- or multi-species microorganisms, biofilm thickness and structures, contact surfaces and food environments. A better understanding of these intrinsic and extrinsic parameters enables the application of PDI to be highly efficient. In addition, researchers are encouraged to investigate hurdle strategies that maximise the synergistic effects.

PDI employment for combating biofilms in the food industry has increased due to its outstanding effectiveness in deconstructing or removing biofilm contamination. While some studies have been published demonstrating the potential of PDI, the antibiofilm mechanisms and influencing factors remain knowledge gaps. The limitations of the PDI technique remain, i.e., visible light’s limited penetration depth limits the conditions under which it can be used. The combined treatments remain unexplored and should be investigated further. Moreover, most studies have been conducted in the laboratory hence more research is needed to scale up for industrial applications. This review provides a firm foundation for the ongoing study of PDI, and paves the way for increased understanding and optimization of biofilm problems in a variety of food environments.

## Figures and Tables

**Table 1:** Applicable photosensitizers (PSs) in food environments.

PS Category	PSs	References
Microbial intercellular components	porphyrins, flavins, cytochromes	[23]
Plant source	Tetrapyrrole macrocycles (chlorins, phthalocyanines and bacteriochlorin), curcumin, hypericin, hematoporphyrin	[25,26]
Other natural PSs	Riboflavin (vitamin B2), Vitamin K3, vitamin D	[27,28,29]
Synthetic chemical	Rose bengal, erythrosine, TiO_2_	[30]

**Table 2:** Applications of photodynamic inactivation (PDI) in food and their effects on food quality.

Food Products	Strains	PS	Light (Dose)/Temperature	Food Quality	Reference
Apricots (*Prunus armeniaca*), plums (*Prunus domestica*) and cauliflower (*Brassica oleracea*)	*Bacillus cereus*	Hypericin	585 nm (6.912 J/cm^2^)	Reached microbial reduction of 1.1, 0.7, 1.3 log_10_ CFU/g on apricots, plums and cauliflower surfaces; No significant changes in antioxidant content.	[35]
Orange juice	*Salmonella* spp.	-	460 nm (4500 J/cm^2^)	Color might change significantly due to over-lighting and overheating. Irradiance of 92.0 mW/cm^2^ resulted in colour threshold values of 500 J/cm^2^ at 4, 12, and 20 °C; irradiance of 254.7 mW/cm^2^ resulted in 1000, 2000, 1000 mW/cm^2^; irradiance of 147.7 mW/cm^2^ resulted in 3500, 500, 2500 mW/cm^2^.	[36]
Milk	*Escherichia coli*	-	405–460 nm/5–15 °C	No significant differences (*p* > 0.05) were detected in moisture, fat, protein, carbohydrate, acidity, pH and viscosity; Minimum overall colour change was with PDI treatment at 406 nm, 13.8 °C, and for 37.83 min.	[37]
Cooked oyster	*Vibrio parahaemolyticus biofilm*	100.0 μM curcumin	405 nm (9.36 J/cm^2^)	Irradiation achieved inactivation to an undetectable level from 5.2 log_10_ CFU/g.	[40]
Maize kernels (Fruit corn)	*Aspergillus flavus*	25 μM Curcumin	400–700 nm (60 J/cm^2^)	2.2 log_10_ CFU/mL reduction of *Aspergillus flavus* spores.	[41]
Grape	*E. coli*	Curcumin	465 nm (36.3 J/cm^2^)	Vitamin C was decreased by 5% of PDI treated samples after 4 days, and 24% decrease of untreated samples; Firmness loss was delayed (28% loss of PDI treated samples and 56% loss of untreated), reduced weight loss (14% loss of PDI treated samples and 38% loss of untreated); No significant colour changes after 4 days storage of PDI samples; 2.4 log_10_ CFU/g viable cell reduction of PDI treated samples.	[42]
Chicken	*Listeria monocytogenes*	Curcumin	430 nm (6.4, 32.1, and 64.2 kJ/m^2^)	There were no recognizable differences in chicken skin colour after treatment with light dose greater or equal to 32.1 kJ/m^2^.	[43]
Packaged sliced cheese	*L. monocytogenes, Pseudomonas fluorescens*	-	460–470 nm/4–25 °C	At 4 °C for 1 h to 7 d of PDI treatment, no significant colour changes were observed, however, increasing irradiation time at ambient temperatures could induce colour differences.	[44]
Fresh-cut mango	*E. coli*, *L. monocytogenes*, *Salmonella* spp.	-	405 nm/4, 10, 20 °C	No significant differences in colour, ascorbic acid, antioxidant capacity, flavonoid and β-carotene between treated and untreated samples regardless of storage temperatures.	[31]
Oyster	*Norovirus*	Curcumin	470 nm (3.6 J/cm^2^)/10 °C	The curcumin under the concentration of 10 μM did not affect colour and flavour while applied in oyster.	[45]

**Table 3:** Antibiofilm activities of PDI on food products.

Food Products	PS	Wavelength	Temperature	Total Dose	Log Reduction	Reference
Pepper	50 μM curcumin bound to polyvinylpyrrolidone	435 nm	Not specified	33.8 J/cm^2^	Killed almost all *Staphylococcus aureus attached colonies* (99.7%).	[49]
Fresh salmon	Endogenous PS	405 nm	4, 12 °C	460.8 J/cm^2^	0.3–0.5 log_10_ CFU/cm^2^ reduction of *L. monocytogenes* and *Salmonella* spp.	[51]
UHT whole milk	TiO_2_	400 nm	Room temperature	Not specified	The *L. monocytogenes* biofilm growing on the glass with nanoparticle (glass surface modified by 16% *w*/*v* TiO_2_) was found to have decreased by 3 log_10_ CFU/cm^2^ after 90 min irradiation.	[50]
Mandarin	Endogenous PS	448–655 nm	20 °C	Not specified	The biofilm formation of *Bacillus amyloliquefaciens* JBC36 decreased significantly by combining red and green light together at an intensity of 240 μM/m^2^s.	[52]

**Table 4:** Antibiofilm mechanisms and genotypic expression changes of PDI.

Bacteria	PDI Treatment	Antibiofilm Mechanisms and Genotypic Expression	Reference
Methicillin-Resistant *S. aureus*	415 nm LED 0–4 J/cm^2^ 20 °C	Rapid generation of ROS; Induced lipid peroxidation; Membrane damage;	[64]
*L. monocytogenes*, *P. fluorescens*	460–470 nm LED 0–604.8 J/cm^2^ 4, 25 °C	Membrane damage; Loss of cytoplasmic components; Metabolic inhibition (RNA, protein, and peptidoglycan metabolism);	[44]
*Salmonella enteritidis*, *Salmonella saintpaul*	405 nm LED 72 J/cm^2^ 4 °C	Membrane damage; Nucleotide oxidation and degradation; Genes (*oxyR*, *recA*, *rpoS*, *sodA*, and *soxR*) were lower expressed after PDI illumination except *oxyR* (Flagellar motor gene) and *recA* (Induces a cellular response to DNA damage-SOS response) were of higher expression levels in *S. Enteritidis* cells.	[65]
*V. parahaemolyticus* biofilm	455–460 nm LED 20.0 μM curcumin13.68 J/cm^2^ 7–37 °C	Cell wall damage; Genomic DNA damage and protein degradation; Decrease in carbohydrate C-O-C group, ring breathing tyrosine, Amide III phenylalanine of proteins and the guanine (G), cytosine (C) and uracil (U) of DNA/RNA of biofilm matrix. The expression levels of flagellar motor gene (*oxyR*) and quorum sensing related genes were down-regulated.	[39]
*Cronobacter sakazakii* biofilm	405 nm LED 26 mW/cm^2^, 2 h 25 °C	Destruct and degrade polysaccharide and protein in the biofilm matrix; A significant reduction of all eight genes (*bcsA*, *bcsG*, *flgJ*, *motA*, *motB*, *luxR*, *fliD*, *flhD*) related to biofilm formation, the greatest difference is the expression of *fliD* gene.	[54]
*L. monocytogenes* biofilm	455–460 nm LED 0.5 μM curcumin 3.24 J/cm^2^ 4–37 °C	Cytoplasmic DNA and protein damage; Reduced adhesion; Deconstruct biofilm architecture; The virulence genes including *inlA*, *hlyA*, and *plcA* significantly down-regulated. The *prfA* responsible for stress response were upregulated.	[26]
*P. aeruginosa* biofilm	650 nm diode laser 0.012 mM methylene blue (MB) 23 J/cm^2^	Disrupted biofilm matrix; Reduced cell clusters; The downregulation of the expression of QS-controlled biofilm formation genes (*pslA* and *pelF*) and QS genes (*lasI*, *lasR*, *rhlI*, and *rhlR*) in *P. aeruginosa* ATCC 27853.	[66]
*Salmonella enterica biofilm*	405 nm LED, 0.015 mM Chl, 38 J/cm^2^	Generation of ROS, especially singlet oxygen; Cell membrane disintegration; Leakage of DNA and protein components; Shrinkage of cells; Genes for the adaptation and protection against oxidative stress (*oxyR*, *ahpC*, *grxA*, *atpC*) and SOS response gene *sulA* were up-regulated.	[67]

**Table 5:** Summarization of potential hurdle strategies accompanying PDI.

Hurdle Strategy	Mechanisms	Advantages & Disadvantages
Organic acids	Undissociated form of organic acids that easily penetrate cell membrane and decrease intracellular pH; Chelation effect of organic acids, and they can disrupt structures of cell membrane; ALA can promote endogenous PS synthesis and accumulation.	Advantages: wide range disinfection, safe and effective; Disadvantages: occurrence of acid resistant and tolerant microorganisms; application requirement of pH less than 5.5; acid ionization will reduce antimicrobial activities [113].
Nanobubble	It expands space between sessile cells, allowing to own radical and intensive energy.	Advantages: long stay time, low energy required, far-reaching force, applicable in liquid disinfection; Disadvantages: easy to be deformed.
Ultrasound	High pressure, high energy, strong agitation, shear stress, and turbulence can kill microorganisms and deconstruct biofilm structures.	Advantages: no ionizing radiation, simple to be applied; Disadvantages: ultrasound can cause adverse effect on food properties like colour or phenolic compounds loss; it also can induce resistant microorganisms [116].
Biosurfactant	It inhibits microbial adherence and biofilm formation.	Advantages: it can inhibit microbial adherence on equipment surfaces and make it easy to proceed hygiene procedure; Disadvantages: should design surfactant depending on different surfaces and food composition in case of compromising the surfactant and contamination of food products.
EDTA	EDTA can chelate the divalent cation which are essential to stability and maintenance of the biofilm matrix, and thus achieve biofilm deconstruction and removal.	Advantages: approved by FDA, it can be used to promote stability of colour, flavour and texture; Disadvantages: it induces long-term lead poisoning [117].
Nanocarrier	Nanocarrier assists in high delivery of PS into biofilm matrix, inhibition of PS degradation, moderate release rate and resultant production of ROS from PDI process.	Advantages: it can control PS degradation and release, and promote ROS production; Disadvantages: in complexity to make sure nanocarrier can work and will not affect food qualities and human health.
Essential oils	Essential oils can modulate antibiotic activities of some bacterium; some essential oils disrupt lipid structure, cause cytoplasmic leakage and lead to cell lysis.	Advantages: natural, safe and friendly; Disadvantages: some essential oils will interact with emulsifiers or food compounds, and it will decrease antimicrobial and antibiofilm activities; essential oils may have pungent odour [118].
Chemical disinfectants	Depending on various disinfectants: Sodium hypochlorite can kill microorganisms and deconstruct biofilms; Electrolyzed water involve disinfection of intracellular potassium leakage, TTC-dehydrogenase relative activity and bacterial ultrastructure destruction; Cold plasma treated water can produce ROS and RONS that kill microorganisms and decontaminate biofouling.	Advantages: widely used disinfectants are easy access and handling, always compatible with other detergents, some disinfectant efficiencies vary in different food environments (pH, temperature, different food matrices); Disadvantages: most are corrosive, by-products from some disinfectant like chlorinated ones will produce chlorine and be risky for food and human health [119].

## Data Availability

Not applicable.
